# Prognostic role of IL-8 in cancer patients treated with immune checkpoint inhibitors: a system review and meta-analysis

**DOI:** 10.3389/fonc.2023.1176574

**Published:** 2023-08-09

**Authors:** Dan Zou, Ailin Song, Wei Yong

**Affiliations:** ^1^ Thyroid & Breast Surgery, Chengdu Seventh People’s Hospital, Chengdu, Sichuan, China; ^2^ Thyroid & Breast Surgery, Lanzhou University Second Hospital, Lanzhou, Gansu, China

**Keywords:** interleukin 8, immune checkpoint inhibitors, prognostic role, biomarkers, meta-analysis

## Abstract

**Background:**

Immune checkpoint inhibitors (ICIs) have been proven to be an effective treatment strategy for a variety of malignant tumors. However, only a subset of patients can benefit from ICIs due to factors such as drug resistance. Therefore, it is crucial to identify biomarkers that can accurately predict the efficacy of ICIs and provide a basis for individualized immunotherapy. In this study, we conducted a systematic review and meta-analysis to explore whether the chemokine interleukin 8 (IL-8) can be used as a biomarker to evaluate the efficacy of ICIs treatment.

**Methods:**

We conducted a comprehensive search of several databases, including PubMed, Embase, Web of Science, and Cochrane, to identify relevant articles published up to June 08, 2023. Our inclusion criteria were limited to cohort studies and clinical trials that reported hazard ratios (HR) and 95% confidence intervals (CI) for overall survival (OS) and/or progression-free survival (PFS), as well as the objective response rate (ORR), in cancer patients with high and low IL-8 expression. For data analysis, we used Revman to generate forest plots, subgroup analysis, and assess publication bias. Additionally, Stata was utilized for sensitivity analysis and further examination of publication bias.

**Results:**

A total of 24 datasets, involving 3190 participants, were selected from 14 studies. The meta-analysis revealed a reduction in ORR, OS, and/or PFS in the high IL-8 group after treatment with ICIs compared to the low IL-8 group.

**Conclusion:**

IL-8 can serve as a biomarker for predicting the efficacy of ICIs. Patients with lower expression of IL-8 may benefit from ICIs treatment.

**Systematic review registration:**

https://www.crd.york.ac.uk/PROSPERO/display_record.php?RecordID=383188, identifier CRD42022383188.

## 1 Introduction

In recent years, immunotherapy has emerged as a viable treatment option for various cancers, particularly through the utilization of monoclonal antibody-based immune checkpoint blockade (ICB). To date, all approved immune checkpoint inhibitors (ICIs) are monoclonal antibodies that block cytotoxic T-lymphocyte-associated protein 4 (CTLA-4), programmed cell death protein 1 (PD-1), or programmed death ligand 1 (PD-L1). These molecules serve as key inhibitors of T-cell activation and function (1, 2). Since ipilimumab was approved by the FDA for metastatic melanoma in 2011, various ICIs have been introduced, Numerous studies have demonstrated the benefits of ICIs in non-small cell lung cancer (3, 4), liver cancer (5), triple-negative breast cancer (6), gastroesophageal cancer (7), ovarian cancer (8) and other malignant tumors. Therefore, ICIs such as nivolumab, ipilimumab, pembrolizumab and atezolizumab have been approved for the treatment of a variety of malignant tumors. Nevertheless, a subset of patients does not have a response to treatment with ICIs or many patients who initially respond eventually experience relapse due to acquired resistance (9). Early identification of patients who are not responsive to ICIs treatment can help avoid ineffective treatment and mitigate potential serious adverse effects. Biomarkers used for predicting response to ICIs treatment encompass PD-L1 expression, mismatch repair deficiency (dMMR), microsatellite instability (MSI), somatic DNA mutation count and/or tumor mutation burden (TMB), indicators of T-cell infiltration, gut microbiota composition, and deleterious somatic variants (10, 11). However, the clinical implementation of these markers poses challenges, and there is a need for improved clinical biomarkers to facilitate the rational selection of appropriate immunotherapy.

Interleukin-8 (IL-8), commonly referred to as the neutrophil chemokine, is secreted by various cell types, such as monocytes, neutrophils, epithelial cells, fibroblasts, endothelial cells, mesothelial cells, and tumor cells (12). IL-8 plays a significant role in various biological processes. It contributes to inflammation and wound healing in non-pathogenic environments and possesses the ability to recruit T cells and non-specific inflammatory cells to the site of inflammation by activating neutrophils (13). Secondly, tumor-derived IL-8 promotes epithelial-mesenchymal transition (EMT) by influencing the tumor microenvironment, thereby facilitating tumor cell migration and invasion. Furthermore, IL-8 stimulates tumor angiogenesis, thereby contributing to tumor progression (13). A number of studies have confirmed that the level of IL-8 in tumor tissues or blood of patients with different cancers is higher compared to those in paracancerous tissues or control groups. Additionally, patients with high expression of IL-8 in tumor tissues or blood tend to have a poor prognosis (14–16). IL-8 is expected to be a biomarker for predicting the prognosis of patients with various cancers.

In recent years, with the approval of ICIs for a variety of malignant tumors, the search for biomarkers to predict the efficacy of ICIs has gained significant attention. Multiple studies have found that higher expression of IL-8 in the blood of patients with non-small cell lung cancer, melanoma, triple-negative breast cancer, head and neck squamous cell carcinoma, and urothelial carcinoma is associated with a poorer benefit in terms of OS and/or PFS following anti-PD-1, anti-PD-L1, or anti-CTLA-4 treatment (17–26). In this study, we conducted a systematic review and meta-analysis of existing clinical studies to evaluate whether IL-8 can be used as a potential biomarker to predict the survival of patients treated with ICIs, explore the predictive efficacy of IL-8 in a variety of tumor types, and provide a foundation for the individualized application of ICIs.

## 2 Methods

The protocol was registered on the International Prospective Register of Systematic Reviews (registration number: CRD42022383188).

### 2.1 Literature research

Pubmed, Embase, Web of science, and Cochrane database were searched for articles published up to June 08, 2023. The keyword was set to “immune checkpoint inhibitors [MeSH]” OR “immune checkpoint block” OR “ICI” OR “PD-1” OR “PD-L1” OR “PD-1/PD-L1” OR “anti-PD-1/anti-PD-L1” OR “CTLA-4”, “pembrolizumab” OR “avelumab” OR “nivolumab” OR “durvalumab” OR “tremelimumab” OR “atezolizumab” OR “immunotherap*” AND “Interleukin-8[MeSH]” OR “CXCL8” OR “Interleukin 8” OR “IL-8” OR “C-X-C Motif Chemokine 8” OR “C-X-C Motif Chemokine Ligand 8” OR “IL8”.

### 2.2 Selection criteria

Inclusion criteria: Patients in the study had a pathologically confirmed solid tumor. Patients had received treatment with at least one immune checkpoint inhibitor during the study. The purpose of this study is to explore the prognostic value and efficacy of IL-8 in solid tumors, and the study should include the HR and 95%CI of OS and/or PFS related to IL-8 level.

Exclusion criteria: basic research or animal experiments related to IL-8, or detection of IL-8 in tissues. Studies with incomplete data or lack of full text. Conference abstracts, reviews, meta-analyses, comments or letters, case reports.

### 2.3 Data extraction and quality assessment

All the literatures retrieved in the four databases with the above search terms were imported into EndNote X9 literature manager to remove duplicate literatures. Two researchers screened the literature according to the inclusion and exclusion criteria. The following information was extracted: first author, publication year, country, study type, tumor type, immunotherapy drug name, IL-8 assay method, sample source, sample size, and corresponding HR and 95% CI of OS and PFS.

The meta-analysis was performed in accordance with the Preferred Reporting Items guidelines of the Systematic Review and Meta-analysis Protocol (PRISMA-P) 2015 statement (27, 28). The Newcastle-Ottawa Quality Assessment Scale (NOS) checklist was utilized by our team of authors to evaluate the quality of the studies included in the analysis. The assessment tool, which focuses on eight items grouped into three categories (selection, comparability, and outcome), assigns a maximum of 9 stars. Articles that scored six or more stars were deemed high quality. Our team conducted the quality assessment independently (29).

### 2.4 Statistical analysis

Hazard ratios (HR) including 95% confidence intervals (CI) were used to assess the association between IL-8 expression levels and OS and PFS in patients with solid tumors treated with ICIs. The observed HR > 1 implies worse prognostic significance in the group with high IL-8 expression. In contrast, HR < 1 implies better prognostic significance in the group with high IL-8 expression. For ORR, OR > 1 showed that the low IL-8 group had better therapeutic effects than the high IL-8 group. Revman 5.3 software (RevMan, The Cochrane Collaboration) was used to assess heterogeneity between studies with the Cochrane Q test and P values. Estimates of HR were weighted and pooled using a Mantel-Haenszel fixed-effects model. Stata 12.0 software (Stata, College Station) was used to assess study sensitivity and publication bias. Publication bias was assessed by Egger’s test, and P < 0.05 was considered statistically significant.

## 3 Results

### 3.1 Characteristics of the included studies and data quality

**Figure 1:**
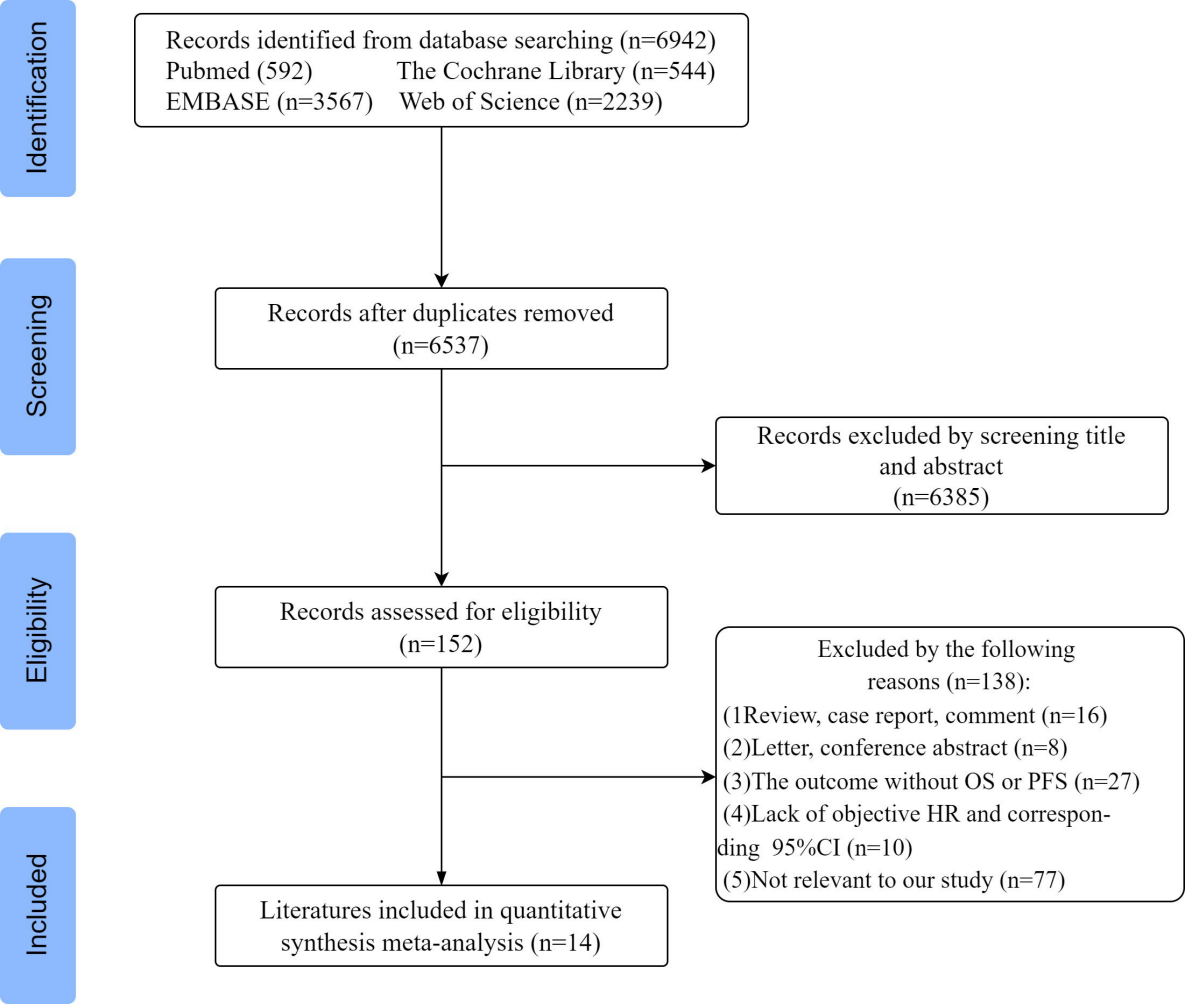
Flowchart of study selection.

According to the above search criteria, 6942 potential studies were preliminarily retrieved in 4 databases, and 6537 were left after removing duplicates. After reviewing the titles and abstracts, based on the inclusion and exclusion criteria, 6385 papers were excluded, leaving 152 papers for further evaluation. Among them, 138 articles were excluded for the following reasons: 16 were reviews and comment cases, 8 were letters and conference abstracts, 27 did not report PFS and OS outcomes, and 10 lacked HR and 95% confidence interval. Additionally, 77 articles were deemed irrelevant to our study. Finally, a total of 14 articles were included for systematic review and meta-analysis. A flowchart of the study selection process is shown in **Figure 1**. In total, 24 datasets were included, with one study using two datasets, one study using five datasets, and one study using six datasets.

A total of 14 studies involving 3272 participants were published between 2017 and 2023, with patient numbers ranging from 16 to 443 in each study. These studies were conducted in eight different countries across North America, Asia, and Europe. The country with the largest number of publications and sample size was the United States. The included studies comprised five clinical trials and nine cohort studies, encompassing various tumor types such as lung cancer, breast cancer, melanoma, urologic tumors, prostate cancer, and head and neck squamous cell carcinoma. Immunotherapy involved the utilization of various immune checkpoint inhibitors (ICIs), including anti-PD-1, anti-PD-L1, anti-CTLA-4, among others.

**Figure 2:**
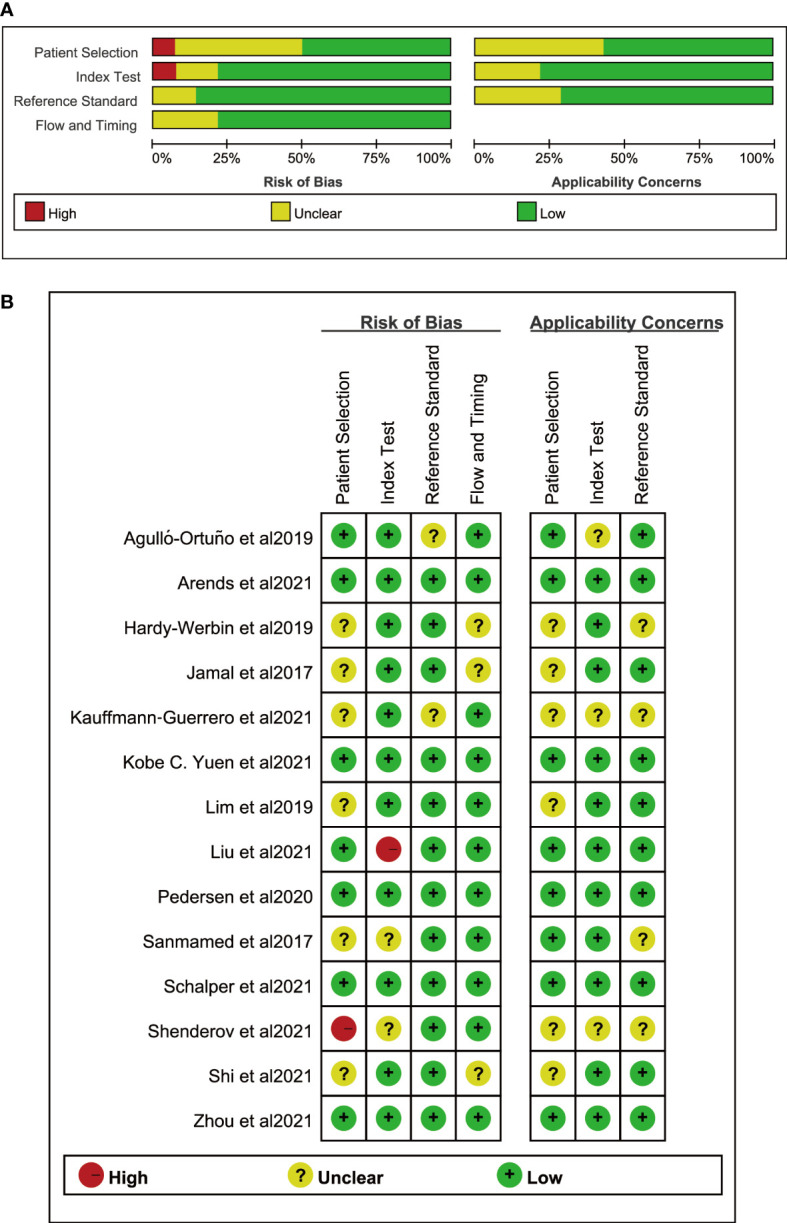
Risk of bias. **(A)** Risk of bias graph. **(B)** Risk of bias summary.

Among the 14 studies, 8 criteria from the NOS quality assessment tool were used to evaluate the quality of the literature. The average quality score of the literature was >6, indicating that the quality of the literature was good. Regarding adaptability, the risk level of all the literature sources was low, and the included sources were deemed to have good adaptability. **Table 1** presents the basic characteristics, details, and NOS scores of the 14 sources included in the study, while **Figure 2** illustrates the scores of each dimension.

**Table 1:** Characteristics of the included studies.

Study	Year	Country	Study Type	Tumor Type	Immunotherapy Drug	Sample Source	IL-8 Detection Method	Cut-off (pg/mL)	No.Patients		Outcome	NOS
									H	L		
Liu et al	2021	China	clinical trial	TNBC	Camrelizumab	blood	Cytokine Factor Panel	NR	12	13	PFS, OS	8
Agulló-Ortuño et al	2019	Spain	cohort study	NSCLC	Nivolumab	blood	ELISA	median value	Total 27		PFS, OS	9
Sanmamed et al	2017	USA	cohort study	melanoma	Nivolumab	blood	ELISA	9.2% change	Total 29		OS	8
				NSCLC	Pembrolizumab				Total 19		
Schalper et al	2020	USA	clinical trial	melanoma	Nivolumab	blood	immunoassay	23	83	209	PFS, OS, ORR	8
				melanoma	Ipilimumab			23	81	217	PFS, OS, ORR
				melanoma	Nivolumab+Ipilimumab			23	84	213	PFS, OS, ORR
				RCC	Nivolumab			23	123	269	PFS, OS, ORR
				sqNSCLC	Nivolumab			23	37	71	PFS, OS, ORR
				nsqNSCLC	Nivolumab			23	78	177	PFS, OS, ORR
Yuen et al	2021	USA	clinical trial	mUC	Atezolizumab	blood	immunoassays	15	Total 88		OS	8
				mUC	Atezolizumab			15	Total 241		OS
				mRCC	Atezolizumab			15	NA		OS
				mRCC	Atezolizumab+Bevacizumab			15	Total 443		OS
				mUC	Atezolizumab			15	NA		OS
Zhou et al	2021	China	cohort study	lung cancer	Anti-PD-1/Anti-PD-L1	blood	NR	7	10	32	OS	7
Jamal et al	2017	Canada	cohort study	melanoma	Ipilimumab	blood	multiplex assay	76	Total 30		OS	9
Hardy-Werbin et al	2019	Spain	ccohort study	small cell lung cancer	Ipilimumab	blood	Luminex assay	13.82	10	27	PFS, OS	8
Shi et al	2021	China	cohort study	NSCLC	Anti-PD-1	blood	EasyMagPlex Human Cytokine 12 Plex Kit	4.9	30	29	PFS, OS	6
Kauffmann-Guerrero et al	2021	Germany	cohort study	NSCLC	Nivolumab/Pembrolizumab	blood	Human Cytokine-Inflammation (9-plex)	19.6	Total 29		PFS	9
Arends et al	2021	USA	clinical trial	HNSCC	Durvalumab	blood	Luminex xMAP technology	median value	73	85	OS	7
Lim et al	2019	Australia	cohort study	melanoma	Anti-PD-1/Anti-CTLA-4	blood	65-plexHuman Cytokine/Chemokine Discovery Assay	NR	Total 58		OS	7
Shenderov et al	2021	USA	clinical trial	prostate Cancer	Nivolumab+ Ipilimumab	blood	ELISA	NR	Total 24		OS, PFS	8
Pedersen et al	2020	Denmark	cohort study	melanoma	Pembrolizumab Ipilimumab/Nivolumab	blood	high-sensitive 10-cytokine U-plex panel	NR	8	8	PFS	7

TNBC, triple-negative breast cancer; NSCLC, non-small cell lung cancer; sqNSCLC, squamous NSCLC; nsqNSCLC, nonsquamous NSCLC; RCC, renal-cell carcinoma; mUC, metastatic urothelial carcinoma; mRCC, metastatic RCC; HNSCC, head and neck squamous cell carcinoma; ELISA, enzyme linked immunosorbent assay; NR, not reported; PFS, progression-free survival;OS, overall survival; ORR, objective response rate.

### 3.2 Comparison of ORR, OS and PFS between high and low IL-8

**Figure 3:**
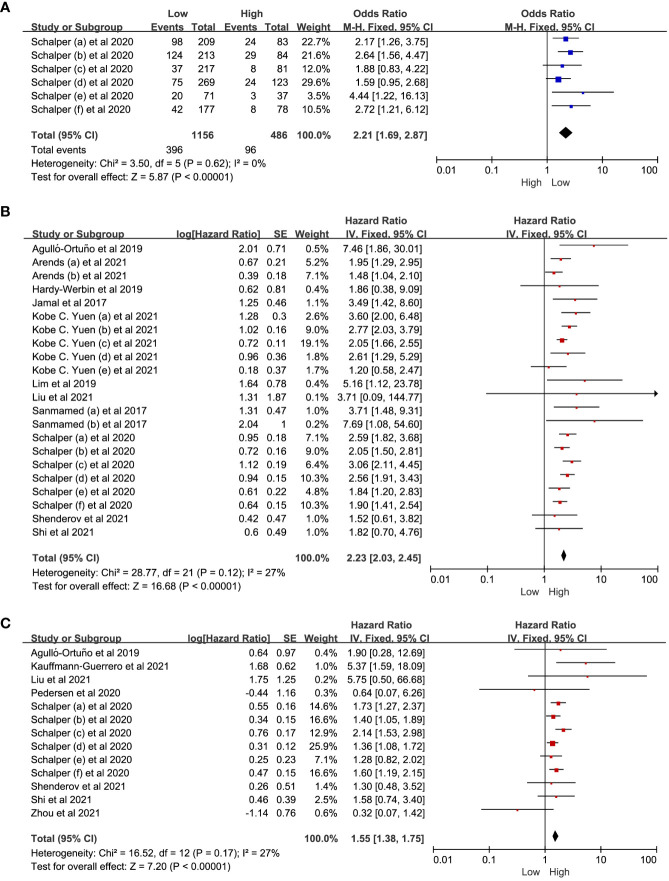
The forest plot of ORR, OS and PFS. **(A)** The forest plot of ORR in patients with low IL-8 *vs.* high IL-8. **(B, C)** The forest plot of OS and PFS in patients with high IL-8 *vs.* low IL-8. ORR, objective response rate; OS, overall survival; PFS, progression-free survival; HR, hazard ratio; CI, confidence interval. The hazard ratios value with 95% confidence intervals generated by Cox proportional hazards model were pooled using the generic inverse variance method for between-groups analysis of OS and PFS. The observed HR > 1 implies worse prognostic significance in the group with high IL-8 expression. In contrast, HR < 1 implies better prognostic significance in the group with high IL-8 expression.

We conducted a meta-analysis of studies investigating on the relationship between IL-8 levels and the ORR, OS and PFS in patients with solid tumors treated with ICIs. The meta-analysis indicated that low IL-8 levels were associated with a better ORR (OR = 2.21, 95% CI: 1.69-2.87, P<0.01). Considering that there was no significant heterogeneity among the studies with OS (I^2^ = 27%, P=0.12), a fixed effect model was used for meta-analysis. The pooled effect HR for OS was 2.23 (95%CI: 2.03-2.45, P<0.01). Similarly, as there was no significant heterogeneity among the studies for PFS (I^2^ = 27%, P=0.17), the fixed effect model was applied, and the pooled effect HR for PFS was 1.55 (95%CI: 1.38-1.75, P<0.01). The results demonstrated that the low-IL-8 group receiving ICIs exhibited significantly greater treatment benefit compared to the high-IL-8 group (**Figures 3A–C**).

### 3.3 Subgroup analyses

**Figure 4:**
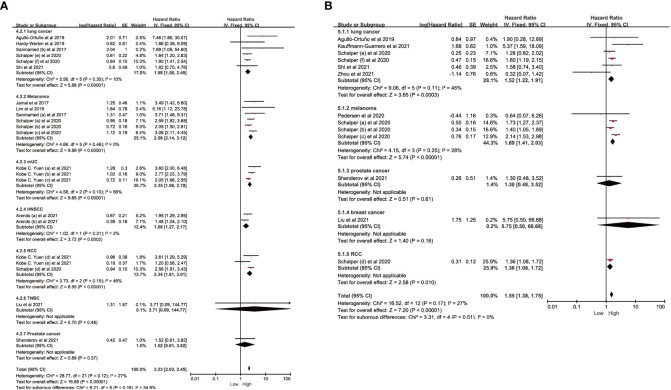
Subgroup analysis in OS and PFS of patients with high *vs.* low IL-8. **(A)** Subgroup analysis in OS of patients with high/low IL-8 based on different cancer types. **(B)** Subgroup analysis in PFS of patients with high/low IL-8 based on different cancer types. OS, overall survival; PFS, progression-free survival; HR, hazard ratio; CI, confidence interval.

To investigate the impact of various factors, including tumor types, race, study types, drug types, and sample size, on the association between IL-8 and prognosis, and to assess whether heterogeneity was influenced by these factors, we conducted subgroup analysis (**Figure 4**; **Tables 2**, **3**). The results indicate that the predictive ability of IL-8 levels for OS and PFS remained consistent across various tumor types. Patients with solid tumors and high IL-8 levels who received ICIs exhibited poorer OS and PFS (**Figures 4A, B**). Regarding the study types, there was no significant difference in heterogeneity between clinical trials and cohort studies. In the subgroup analysis based on race, we observed that Western individuals with high IL-8 levels had worse OS compared to Eastern individuals. However, the predictive value of IL-8 levels for PFS remained consistent across different races. There are multiple types of ICIs approved by the FDA, and the results consistently demonstrate that IL-8 levels predict OS in patients receiving different ICIs. Heterogeneity analysis showed significant differences between anti-PD-L1 groups. In addition, we divided the sample size into > 30 large sample group and ≤30 small sample group according to the size of the sample, and the sample size had no significant effect on the source of study heterogeneity.

**Table 2:** Subgroup of OS.

Subgroup	Heterogeneity				
	No of studies	I^2^	P values	Pooled HR (95% CI)	P values
Study type					
Clinical trail	15	32%	0.11	2.18 [1.98, 2.41]	<0.01
Cohort study	7	0%	0.62	3.41 [2.19, 5.31]	<0.01
Race					
Easterner	2	0%	0.71	1.91 [0.75, 4.83]	0.17
Westerner	20	33%	0.07	2.23 [2.03, 2.45]	<0.01
Immunotherapy drug					
anti-PD-1	9	12%	0.33	2.31 [1.97, 2.72]	<0.01
anti-PD-L1	7	53%	0.04	2.10 [1.83, 2.41]	<0.01
anti-CTLA-4	3	0%	0.54	2.16 [1.62, 2.90]	<0.01
anti-PD-1+anti-CTLA-4	3	20%	0.29	2.86 [2.04, 4.01]	<0.01
sample size					
≤30	6	0%	0.44	3.26 [2.03, 5.24]	<0.01
>30	16	30%	0.12	2.20 [2.00, 2.42]	<0.01

**Table 3:** Subgroup of PFS.

Subgroup	Heterogeneity				
	No of studies	I^2^	P values	Pooled HR (95% CI)	P values
Study type					
Clinical trail	8	0%	0.48	1.60 [1.40, 1.84]	<0.01
Cohort study	6	45%	0.11	1.62 [1.41, 1.85]	<0.01
Race					
Easterner	4	45%	0.14	1.44 [0.84, 2.45]	<0.01
Westerner	10	9%	0.36	1.62 [1.41, 1.85]	<0.01
Immunotherapy drug					
anti-PD-1	9	0%	0.58	1.61 [1.36, 1.90]	<0.01
anti-PD-1/PD-L1	1	NA	NA	0.32 [0.07, 1.42]	0.13
anti-CTLA-4	1	NA	NA	1.40 [1.05, 1.89]	0.02
anti-PD-1+anti-CTLA-4	3	0%	0.4	1.99 [1.46, 2.72]	<0.01
sample size					
≤30	5	12%	0.37	2.04 [1.05, 3.99]	<0.01
>30	9	19%	0.28	1.58 [1.39, 1.81]	<0.01

### 3.4 Sensitivity analysis and publication bias

**Figure 5:**
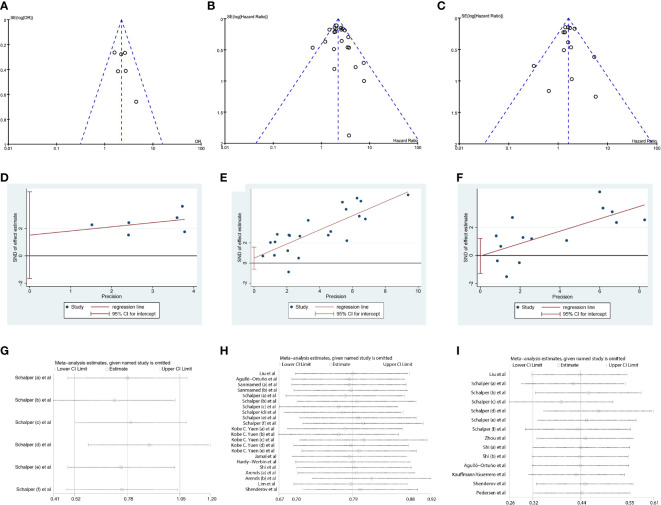
Sensitivity analysis and publication bias. **(A–C)** The funnel plot of studies investigating levels of IL-8 in ORR, OS and PFS. **(D–F)** Egger’s publication bias plots of IL-8 in ORR, OS and PFS. **(G–I)**. Sensitivity analyses of IL-8 in OS and PFS. ORR, objective response rate; OS, overall survival; PFS,s progression-free survival.

The funnel plot showed symmetry (**Figures 5A–C**), and the Egger test revealed p values of 0.255 for ORR, 0.316 for OS and 0.963 for PFS (**Figures 5D–F**), indicating no significant publication bias. Furthermore, sensitivity analyses demonstrated minimal variation in the combined effect estimate, regardless of the excluded study (**Figures 5G–I**).

## 4 Discussion

The application of ICIs for treating solid tumors has demonstrated promising results. However, there are clinical challenges in identifying patients who will benefit from ICIs treatment. Not all patients with solid tumors respond favorably to ICIs, making the prediction of treatment efficacy a significant challenge. Although biomarkers like PD-L1 expression and TMB are commonly used for response prediction, their utility is limited, necessitating the exploration of additional biomarkers (10). The detection methods for different biomarkers vary considerably. For instance, PD-L1 detection involves immunohistochemical staining, and the sensitivity and specificity of this method can significantly impact the accuracy of the results (30). TMB testing is not widely accessible or covered by insurance, potentially limiting patients’ access to these diagnostic tests (31). Furthermore, solid tumors encompass a diverse group of diseases, and the response to ICIs treatment may vary among patients with different tumor types. The heterogeneity of the patient population poses challenges in identifying biomarkers that universally predict treatment response (31). There is currently no standardized protocol for screening patients undergoing treatment with ICIs, leading to variability in patient selection and treatment approaches. Therefore, it is imperative to explore more effective biomarkers to predict the response to immunotherapy in solid tumors and establish an accurate prediction system. This will facilitate the identification of the optimal patient population for ICIs treatment among solid tumor patients.

The progression of cancer is governed not only by the equilibrium between oncogenes and tumor suppressor genes within cancer cells but also by alterations in the tumor microenvironment. The tumor inflammatory microenvironment has garnered significant attention as a crucial factor influencing both the sensitivity of tumor cells to immunotherapy and the prognosis of patients. IL-8, the first chemokine to be identified, is an angiogenic polypeptide expressed in various types of cancer (32). IL-8 effectively regulates the chemotaxis of human neutrophils and exerts direct oncogenic effects, such as promoting angiogenesis, tumor cell dedifferentiation, and facilitating invasion and/or metastasis (33, 34). In recent years, it has been discovered that IL-8 exhibits a dual immune resistance phenomenon. Apart from its direct impact on tumor cells, tumor-derived IL-8 also promotes the recruitment of neutrophils and myeloid-derived suppressor cells (MDSCs) into the tumor microenvironment. These cells have the ability to locally inhibit anti-tumor immune response (35, 36). Schalper et al. found in lung cancer, melanoma, and renal cell carcinoma that circulating IL-8 was positively correlated with CXCL8 gene expression and neutrophil and mononuclear cell counts in tumors. IL-8 levels in tumor tissues were negatively correlated with IFNγ and transcriptional signatures associated with T-cell infiltration, while positively correlated with increased infiltration of myeloperoxidase (MPO)^+^ and/or CD15^+^ monocytes and neutrophils. These data support the adaptive immunosuppressive effect of IL-8 produced by tumor cells (22). Several studies have found that the higher the expression of IL-8 in the blood of patients with a variety of solid tumors, the lower the OS and/or PFS benefit of immunotherapy (21, 22).

To investigate the relationship between IL-8 and solid tumor immunotherapy, we conducted a meta-analysis of 14 studies, evaluating the association between peripheral blood IL-8 levels and the prognosis of patients undergoing solid tumor immunotherapy. Our findings demonstrated that patients with low IL-8 levels after immunotherapy exhibited improved OS, PFS, and ORR compared to those with high IL-8 levels, which aligns with previous studies (37). These results indicate that IL-8 holds promise as a biomarker for immune response, and patients with lower IL-8 levels in tumors may benefit from ICIs treatment.

To investigate the influence of different factors, including tumor types, race, study type, sample size, and drug type, on the relationship between IL-8 and prognosis and to identify the source of heterogeneity, we conducted subgroup analysis. Our findings revealed that patients with high IL-8 levels in solid tumors who received ICIs treatment had a poorer prognosis compared to those with low IL-8 levels. Heterogeneity analysis showed no significant difference between the groups of different tumor types, suggesting that IL-8’s predictive ability as a potential biomarker for immunotherapy efficacy was consistent across various solid tumors. Furthermore, the predictive value of IL-8 for OS varied among different races, with individuals from Western countries exhibiting worse OS when having high IL-8 levels compared to individuals from Eastern countries. However, the relationship between IL-8 and ICIs has only been reported in two studies from China, and the small sample size of these studies may impact the reliability of the results. Despite the diversity of ICIs and their distinct targets, the predictive value of IL-8 for various ICIs in solid tumors remains generally consistent. Apart from race and drug class, study type and sample size did not significantly contribute to the observed heterogeneity

This study is the first to investigate the effect of IL-8 level on the prognosis of patients with solid tumors receiving immunotherapy, and the results of our meta-analysis suggest that IL-8 level may be a potential immunotherapy predictive biomarker in solid tumors. Compared with the existing biomarkers for immunotherapy prediction, serum IL-8 level is a simple quantitative parameter, which can be easily measured in routine blood samples in clinical practice, and can be better applied in clinical practice. However, this study has certain limitations. Firstly, there are insufficient studies on the relationship between ICIs treatment and IL-8 levels in solid tumors; only 14 relevant studies were included in our study, so it is not possible to obtain complete and credible meta-analysis results for all tumor types. Second, not all studies reported all subgroup factors, so in subgroup analyses, effects were pooled only for studies that reported a certain number of subgroup factors, which may lead to inaccurate identification of factors contributing to heterogeneity. Third, some studies have simultaneously used targeted therapy and ICIs, and multiple studies have indicated that IL-8 directly contributes to the resistance of renal cell carcinoma to chemotherapy and molecular targeted drugs (such as VEGF-TKIs) (38). Therefore, the specific predictive ability of IL-8 levels for OS may be influenced by factors such as targeted therapy. Although we analyzed the correlation between IL-8 levels and PFS and ORR, some studies did not provide the original data, which hindered the evaluation of disease control rate (DCR) and duration of response (DOR), thus reducing the reliability of the results. With the rapid development of immunotherapy in the field of tumor treatment, more studies with large samples of IL-8 will be included in the comprehensive analysis in the future, so as to obtain accurate conclusions about the relationship between ICIs and IL-8.

## 5 Conclusion

This systematic review and meta-analysis revealed that patients with high IL-8 levels treated with ICIs had significantly lower ORR, OS, and PFS compared to those with low IL-8 levels. IL-8 levels can serve as a potential prognostic biomarker for cancer patients undergoing ICIs treatment, enabling accurate identification of individuals who would benefit from ICIs. Moreover, it offers new potential treatment strategies for patients with low IL-8 levels who have experienced chemotherapy and targeted therapy failures.

## Author contributions

DZ and WY designed this study. DZ, and AS wrote the manuscript. DZ, and AS participated in the coordination of the study and interpretation of results. DZ, and AS analyzed the data. DZ, WY and AS revised the manuscript. All authors contributed to the article and approved the submitted version.

## References

[1] Moslehi J Lichtman AH Sharpe AH Galluzzi L Kitsis RN . Immune checkpoint inhibitor-associated myocarditis: manifestations and mechanisms. J Clin Invest (2021) 131(5):e145186. doi: 10.1172/jci145186 33645548 PMC7919710

[2] Caron M Lamarre G Grégoire P Simonyan D Laflamme N . The fecal immunochemical test (Fit): selected aspects regarding its effectiveness for colorectal cancer screening in Quebec City. Prev Med Rep (2018) 12:6–11. doi: 10.1016/j.pmedr.2018.08.003 30116704 PMC6082993

[3] Paz-Ares L Ciuleanu TE Cobo M Schenker M Zurawski B Menezes J . First-line nivolumab plus ipilimumab combined with two cycles of chemotherapy in patients with non-small-cell lung cancer (Checkmate 9la): an international, randomised, open-label, phase 3 trial. Lancet Oncol (2021) 22(2):198–211. doi: 10.1016/s1470-2045(20)30641-0 33476593

[4] Reck M Schenker M Lee KH Provencio M Nishio M Lesniewski-Kmak K . Nivolumab plus ipilimumab versus chemotherapy as first-line treatment in advanced non-small-cell lung cancer with high tumour mutational burden: patient-reported outcomes results from the randomised, open-label, phase iii checkmate 227 trial. Eur J Cancer (2019) 116:137–47. doi: 10.1016/j.ejca.2019.05.008 31195357

[5] Finkelmeier F Waidmann O Trojan J . Nivolumab for the treatment of hepatocellular carcinoma. Expert Rev Anticancer Ther (2018) 18(12):1169–75. doi: 10.1080/14737140.2018.1535315 30304963

[6] Kwapisz D . Pembrolizumab and atezolizumab in triple-negative breast cancer. Cancer Immunol Immunother (2021) 70(3):607–17. doi: 10.1007/s00262-020-02736-z PMC10992894 33015734

[7] Shitara K Ajani JA Moehler M Garrido M Gallardo C Shen L . Nivolumab plus chemotherapy or ipilimumab in gastro-oesophageal cancer. Nature (2022) 603(7903):942–8. doi: 10.1038/s41586-022-04508-4 PMC8967713 35322232

[8] Zamarin D Burger RA Sill MW Powell DJ Jr. Lankes HA Feldman MD . Randomized phase ii trial of nivolumab versus nivolumab and ipilimumab for recurrent or persistent ovarian cancer: an nrg oncology study. J Clin Oncol (2020) 38(16):1814–23. doi: 10.1200/jco.19.02059 PMC7255977 32275468

[9] Fares CM Van Allen EM Drake CG Allison JP Hu-Lieskovan S . Mechanisms of resistance to immune checkpoint blockade: why does checkpoint inhibitor immunotherapy not work for all patients? Am Soc Clin Oncol Educ Book (2019) 39:147–64. doi: 10.1200/edbk_240837 31099674

[10] Havel JJ Chowell D Chan TA . The evolving landscape of biomarkers for checkpoint inhibitor immunotherapy. Nat Rev Cancer (2019) 19(3):133–50. doi: 10.1038/s41568-019-0116-x PMC6705396 30755690

[11] Schalper KA Kaftan E Herbst RS . Predictive biomarkers for pd-1 axis therapies: the hidden treasure or a call for research. Clin Cancer Res (2016) 22(9):2102–4. doi: 10.1158/1078-0432.Ccr-16-0169 PMC4940186 26957559

[12] Moser B Loetscher P . Lymphocyte traffic control by chemokines. Nat Immunol (2001) 2(2):123–8. doi: 10.1038/84219 11175804

[13] Gonzalez-Aparicio M Alfaro C . Significance of the il-8 pathway for immunotherapy. Hum Vaccin Immunother (2020) 16(10):2312–7. doi: 10.1080/21645515.2019.1696075 PMC7644160 31860375

[14] Mahmoud MA Ali MH Hassoba HM Elhadidy GS . Serum interleukin-8 and insulin like growth factor-1 in Egyptian bladder cancer patients. Cancer biomark (2010) 6(2):105–10. doi: 10.3233/cbm-2009-0133 PMC12922846 20571236

[15] Konno H Ohta M Baba M Suzuki S Nakamura S . The role of circulating il-8 and vegf protein in the progression of gastric cancer. Cancer Sci (2003) 94(8):735–40. doi: 10.1111/j.1349-7006.2003.tb01511.x PMC11160261 12901801

[16] Rubie C Frick VO Pfeil S Wagner M Kollmar O Kopp B . Correlation of il-8 with induction, progression and metastatic potential of colorectal cancer. World J Gastroenterol (2007) 13(37):4996–5002. doi: 10.3748/wjg.v13.i37.4996 17854143 PMC4434624

[17] Agulló-Ortuño MT Gómez-Martín Ó Ponce S Iglesias L Ojeda L Ferrer I . Blood predictive biomarkers for patients with non-small-cell lung cancer associated with clinical response to nivolumab. Clin Lung Cancer (2020) 21(1):75–85. doi: 10.1016/j.cllc.2019.08.006 31562055

[18] Sanmamed MF Perez-Gracia JL Schalper KA Fusco JP Gonzalez A Rodriguez-Ruiz ME . Changes in serum interleukin-8 (Il-8) levels reflect and predict response to anti-pd-1 treatment in melanoma and non-small-cell lung cancer patients. Ann Oncol (2017) 28(8):1988–95. doi: 10.1093/annonc/mdx190 PMC5834104 28595336

[19] Liu J Li Y Li Q Liang D Wang Q Liu Q . Biomarkers of response to camrelizumab combined with apatinib: an analysis from a phase ii trial in advanced triple-negative breast cancer patients. Breast Cancer Res Treat (2021) 186(3):687–97. doi: 10.1007/s10549-021-06128-4 33634417

[20] Arends R Guo X Baverel PG González-García I Xie J Morsli N . Association of circulating protein biomarkers with clinical outcomes of durvalumab in head and neck squamous cell carcinoma. Oncoimmunology (2021) 10(1):1898104. doi: 10.1080/2162402x.2021.1898104 33796405 PMC7993189

[21] Yuen KC Liu LF Gupta V Madireddi S Keerthivasan S Li C . High systemic and tumor-associated il-8 correlates with reduced clinical benefit of pd-L1 blockade. Nat Med (2020) 26(5):693–8. doi: 10.1038/s41591-020-0860-1 PMC8286544 32405063

[22] Schalper KA Carleton M Zhou M Chen T Feng Y Huang SP . Elevated serum interleukin-8 is associated with enhanced intratumor neutrophils and reduced clinical benefit of immune-checkpoint inhibitors. Nat Med (2020) 26(5):688–92. doi: 10.1038/s41591-020-0856-x PMC8127102 32405062

[23] Zhou J Chao Y Yao D Ding N Li J Gao L . Impact of chronic obstructive pulmonary disease on immune checkpoint inhibitor efficacy in advanced lung cancer and the potential prognostic factors. Transl Lung Cancer Res (2021) 10(5):2148–62. doi: 10.21037/tlcr-21-214 PMC8182718 34164266

[24] Jamal R Lapointe R Cocolakis E Thébault P Kazemi S Friedmann JE . Peripheral and local predictive immune signatures identified in a phase ii trial of ipilimumab with carboplatin/paclitaxel in unresectable stage iii or stage iv melanoma. J Immunother Cancer (2017) 5(1):83. doi: 10.1186/s40425-017-0290-x 29157311 PMC5696743

[25] Hardy-Werbin M Rocha P Arpi O Taus Á Nonell L Durán X . Serum cytokine levels as predictive biomarkers of benefit from ipilimumab in small cell lung cancer. Oncoimmunology (2019) 8(6):e1593810. doi: 10.1080/2162402x.2019.1593810 31069160 PMC6492977

[26] Shi Y Liu X Du J Zhang D Liu J Chen M . Circulating cytokines associated with clinical outcomes in advanced non-small cell lung cancer patients who received chemoimmunotherapy. Thorac Cancer (2022) 13(2):219–27. doi: 10.1111/1759-7714.14248 PMC8758427 34825500

[27] Moher D Liberati A Tetzlaff J Altman DG . Preferred reporting items for systematic reviews and meta-analyses: the prisma statement. Int J Surg (2010) 8(5):336–41. doi: 10.1016/j.ijsu.2010.02.007 20171303

[28] Stroup DF Berlin JA Morton SC Olkin I Williamson GD Rennie D . Meta-analysis of observational studies in epidemiology: A proposal for reporting. Meta-analysis of observational studies in epidemiology (Moose) group. Jama (2000) 283(15):2008–12. doi: 10.1001/jama.283.15.2008 10789670

[29] Stang A . Critical evaluation of the newcastle-ottawa scale for the assessment of the quality of nonrandomized studies in meta-analyses. Eur J Epidemiol (2010) 25(9):603–5. doi: 10.1007/s10654-010-9491-z 20652370

[30] Remon J Besse B Soria JC . Successes and failures: what did we learn from recent first-line treatment immunotherapy trials in non-small cell lung cancer? BMC Med (2017) 15(1):55. doi: 10.1186/s12916-017-0819-3 28285592 PMC5346853

[31] Jardim DL Goodman A de Melo Gagliato D Kurzrock R . The challenges of tumor mutational burden as an immunotherapy biomarker. Cancer Cell (2021) 39(2):154–73. doi: 10.1016/j.ccell.2020.10.001 PMC7878292 33125859

[32] Moore BB Arenberg DA Stoy K Morgan T Addison CL Morris SB . Distinct cxc chemokines mediate tumorigenicity of prostate cancer cells. Am J Pathol (1999) 154(5):1503–12. doi: 10.1016/s0002-9440(10)65404-1 PMC1866583 10329603

[33] Nam K Kimura S Fujiki H Imanishi Y . Effects of phorbol ester and teleocidin on ca2+-induced fusion of liposomes. Biochem Biophys Res Commun (1989) 165(3):1256–61. doi: 10.1016/0006-291x(89)92737-x 2610691

[34] Baggiolini M Walz A Kunkel SL . Neutrophil-activating peptide-1/interleukin 8, a novel cytokine that activates neutrophils. J Clin Invest (1989) 84(4):1045–9. doi: 10.1172/jci114265 PMC329758 2677047

[35] Brat DJ Bellail AC Van Meir EG . The role of interleukin-8 and its receptors in gliomagenesis and tumoral angiogenesis. Neuro Oncol (2005) 7(2):122–33. doi: 10.1215/s1152851704001061 PMC1871893 15831231

[36] David JM Dominguez C Hamilton DH Palena C . The il-8/il-8r axis: A double agent in tumor immune resistance. Vaccines (2016) 4(3):22. doi: 10.3390/vaccines4030022 27348007 PMC5041016

[37] Mao XC Yang CC Yang YF Yan LJ Ding ZN Liu H . Peripheral cytokine levels as novel predictors of survival in cancer patients treated with immune checkpoint inhibitors: A systematic review and meta-analysis. Front Immunol (2022) 13:884592. doi: 10.3389/fimmu.2022.884592 36072577 PMC9441870

[38] Rizzo M Varnier L Pezzicoli G Pirovano M Cosmai L Porta C . Il-8 and its role as a potential biomarker of resistance to anti-angiogenic agents and immune checkpoint inhibitors in metastatic renal cell carcinoma. Front Oncol (2022) 12:990568. doi: 10.3389/fonc.2022.990568 36059687 PMC9437355

